# Revascularization outcomes in diabetic patients presenting with acute coronary syndrome with non-ST elevation

**DOI:** 10.1186/s12933-022-01595-5

**Published:** 2022-09-05

**Authors:** Eilon Ram, Enrique Z. Fisman, Alexander Tenenbaum, Zaza Iakobishvili, Yael Peled, Ehud Raanani, Leonid Sternik

**Affiliations:** 1 Department of Cardiac Surgery, Tel Aviv University, Tel Aviv, Israel; 2 Department of Cardiology, Tel Aviv University, Tel Aviv, Israel; 3 Leviev Cardiothoracic and Vascular Center, Sheba Medical Center, affiliated to the Sackler School of Medicine, Tel Aviv University, Ramat Gan, Israel; 4 The Sheba Talpiot Medical Leadership Program, Ramat Gan, Israel; 5 Clalit Health Services, Tel Aviv, Israel; 6 Sackler Faculty of Medicine, Tel Aviv University, Tel Aviv, Israel; 7 Department of Cardiac Surgery, Sheba Medical Center, Tel Hashomer, 52621 Ramat Gan, Israel

## Abstract

**Background:**

To compare the outcomes of diabetic patients hospitalized with non-ST elevation myocardial infarction (NSTEMI) or unstable angina (UA) referred for revascularization by either coronary artery bypass grafting (CABG) or percutaneous coronary intervention (PCI) in a real-life setting.

**Methods:**

The study included 1987 patients with diabetes mellitus enrolled from the biennial Acute Coronary Syndrome Israeli Survey between 2000 and 2016, who were hospitalized for NSTEMI or UA, and underwent either PCI (N = 1652, 83%) or CABG (N = 335, 17%). Propensity score-matching analysis compared all-cause mortality in 200 pairs (1:1) who underwent revascularization by either PCI or CABG.

**Results:**

Independent predictors for CABG referral included 3-vessel coronary artery disease (OR 4.9, 95% CI 3.6–6.8, p < 0.001), absence of on-site cardiac surgery (OR 1.4, 95% CI 1.1–1.9, p = 0.013), no previous PCI (OR 1.5, 95% CI 1.1–2.2, p = 0.024) or MI (OR 1.7, 95% CI 1.2–2.6, p = 0.002). While at 2 years of follow-up, survival analysis revealed no differences in mortality risk between the surgical and percutaneous revascularization groups (log-rank p = 0.996), after 2 years CABG was associated with a significant survival benefit (HR 1.53, 95% CI 1.07–2.21; p = 0.021). Comparison of the propensity score matching pairs also revealed a consistent long-term advantage toward CABG (log-rank p = 0.031).

**Conclusions:**

In a real-life setting, revascularization by CABG of diabetic patients hospitalized with NSTEMI/UA is associated with better long-term outcomes. Prospective randomized studies are warranted in order to provide more effective recommendations in future guidelines.

**Supplementary Information:**

The online version contains supplementary material available at 10.1186/s12933-022-01595-5.

## Introduction

Ischemic heart disease is highly prevalent in patients with diabetes mellitus, and accounts for more than one-half of all deaths in this population in the USA [1]. In diabetic patients with coronary artery disease (CAD), the superiority of coronary artery bypass grafting (CABG) over percutaneous coronary intervention (PCI) has been demonstrated in several studies world-wide [2, 3], and is the standard treatment of choice and preferred revascularization strategy recommended by the current guidelines (Class IA) [4].

Diabetic patients presenting with acute coronary syndrome (ACS), such as non-ST-segment elevation myocardial infarction (NSTEMI) or unstable angina (UA), have poor prognoses due to multiple comorbidities [5]. Furthermore, they require earlier revascularization than patients with stable CAD [6]. In the setting of ACS without ST-segment elevation, there are no prospective studies devoted entirely to revascularization strategies. As a result, current recommendations regarding the choice of lesions to be treated and treatment modalities are based on parallel findings obtained from stable CAD or STEMI [4].

Current referral patterns and outcomes of diabetic patients hospitalized with NSTEMI/UA referred to either CABG or PCI are unknown. Furthermore, the comparison between the two revascularization strategies is based on cumulative data, which have revealed inconsistent results in various reports [7, 8]. There is therefore a justifiable need to study the real-life results of hemodynamically stable diabetic patients with ACS, who are potential candidates for either one of the revascularization strategies.

## Materials and methods

### Study design

The ACS Israeli Survey (ACSIS) is a voluntary biennial prospective national registry of all patients with ACS hospitalized in the 25 coronary care units and cardiology departments in all the public health hospitals in Israel over a 2-month period (from March to April) [9].

ACSIS is managed by the Working Group on Acute Cardiovascular Care of the Israel Heart Society, in participation with the Israeli Center for Cardiovascular Research. Demographic, historical, and clinical data from all patients were recorded on pre-specified forms. Patient management was at the discretion of the attending physicians. Admission and discharge diagnoses were recorded as determined by the attending physicians based on clinical, electrocardiographic, and biochemical criteria. Definitions of type of MI and UA were homogeneous, based on pre-specified criteria according to accepted definitions during the specific study period [10].

### Study population

**Fig. 1:**
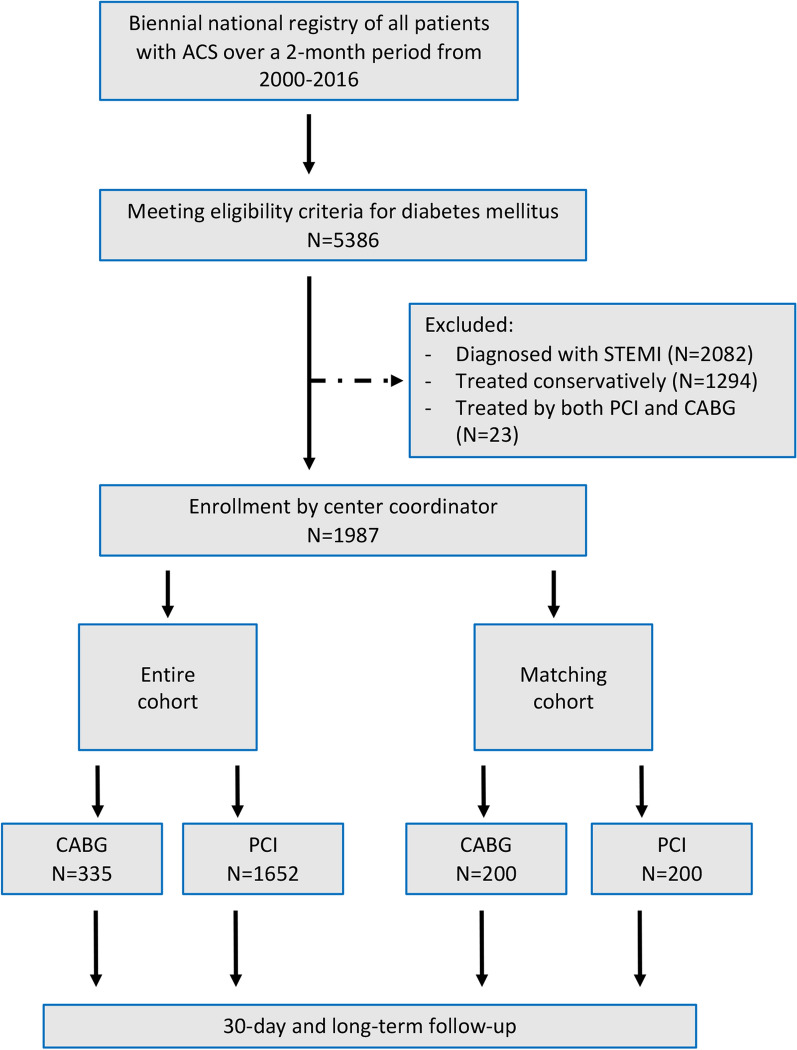
Flow chart summary from eligibility through follow-up. ACS, Acute coronary syndrome; STEMI, ST-elevation myocardial infarction; PCI, Percutaneous coronary intervention; CABG, Coronary artery bypass grafting

Between 2000 and 2016 (which included 8 consecutive registries), 5386 diabetic patients were hospitalized with ACS and were included in the ACSIS registry. Of them, 2087 patients were diagnosed with STEMI, and were excluded from the current study. A further 1312 patients with either NSTEMI or UA were treated either conservatively or by both PCI and CABG, and were therefore also excluded from the current study. Accordingly, the remaining 1987 patients were categorized according to their chosen revascularization strategy: PCI or CABG (Fig. 1). Comparisons were made using data from each of the 8 registries.

### Clinical outcomes

Clinical outcomes included 30-day major adverse cardiac events (MACE: which included death, MI, stroke, and urgent revascularization), in-hospital complications, and long-term all-cause mortality.

### Data collection and follow-up

All data from the 25 participating hospitals were collected and pooled into a designated database. All centers used standardized definitions for data collection, including demographic parameters, medical history, chronic and peri-procedural medical treatment, echocardiography measurements, procedure information and outcome measures. All patients were prospectively followed up for clinical events at 30 days and for late mortality. Mortality data were ascertained from the Israeli Ministry of Interior Population Register through January 2018.

### Ethical statement

All patients in each medical center signed an informed consent form prior to participating in the ACSIS registry, and each center received approval from its institutional review board [11].

### Statistical analysis

Data are presented as mean ± standard deviation for normal, or median for abnormal distribution. Continuous variables were tested with the Kolmogorov–Smirnov test for normal distribution. Categorical variables are given as frequencies and percentages. A chi-square test was used for comparison of categorical variables between revascularization strategies (CABG and PCI), a Student t-test was performed for comparison of normally distributed continuous variables, and a Mann–Whitney U test for non-normal distribution.

To reduce treatment selection bias and potential confounding factors, and to adjust for significant differences in patient characteristics, propensity score-matching was performed. Propensity scores were estimated using a multivariate logistic regression model for treatment with either PCI or CABG. All variables presented in Table 1 were entered to calculate the propensity score. A local optimal algorithm with the caliper method was used for the development of propensity score-matched pairs without replacement (1:1 match). A matching caliper of 0.2 standard deviations of the logit of the estimated propensity score was enforced to ensure that matches of poor fit were excluded. The matching procedure was performed by using the R Matching package. After propensity score-matching, covariates were compared as described above.

**Table 1:** Baseline characteristics

	Before matching			After matching		
	PCI N = 1652 (%)	CABG N = 335 (%)	p-value	PCI N = 200 (%)	CABG N = 200 (%)	p-value
Age (years, mean [SD])	66 (11)	66 (10)	0.517	66 (10)	66 (10)	0.753
Gender (male)	1185 (72)	247 (74)	0.498	135 (67)	146 (73)	0.274
Hypertension	1296 (79)	253 (76)	0.249	158 (79)	151 (75)	0.474
Current smoker	423 (26)	90 (27)	0.654	60 (30)	57 (28)	0.826
Hyperlipidemia	1381 (84)	256 (77)	0.003	167 (83)	159 (79)	0.367
Prior PCI	787 (48)	107 (32)	< 0.001	61 (30)	62 (31)	1.000
Prior MI	736 (45)	104 (31)	< 0.001	68 (34)	64 (32)	0.750
Ejection fraction (%)			0.022			0.765
> 50	566 (50)	117 (41)		95 (48)	95 (48)	
40–50	297 (27)	91 (32)		53 (26)	57 (28)	
30–40	180 (16)	49 (17)		37 (18)	30 (15)	
< 30	79 (7)	29 (10)		15 (8)	18 (9)	
Renal impairment	274 (17)	44 (13)	0.137	38 (19)	34 (17)	0.696
COPD	89 (7)	14 (6)	0.493	13 (9)	8 (6)	0.513
History of CVA/TIA	178 (11)	34 (10)	0.833	18 (9)	18 (9)	1.000
Congestive heart failure	188 (11)	43 (13)	0.492	26 (13)	25 (12)	1.000
Vessels involved			< 0.001			0.901
1-vessel CAD	363 (27)	11 (4)		10 (5)	9 (4)	
2-vessel CAD	454 (34)	53 (21)		46 (23)	43 (22)	
3-vessel CAD	534 (39)	193 (75)		144 (72)	148 (74)	
Indication for angiography			0.810			0.816
NSTEMI	1140 (69)	234 (70)		153 (76)	150 (75)	
Unstable angina	512 (31)	101 (30)		47 (24)	50 (25)	
Time period: after year 2008	1036 (63)	181 (54)	0.004	143 (71)	132 (66)	0.281

CABG, Coronary artery bypass graft; PCI, Percutaneous coronary intervention; SD, Standard deviation; MI, Myocardial infarction; COPD, Chronic obstructive pulmonary disease; CVA, Cerebrovascular accident; TIA, Transient ischemic attack; CAD, Coronary artery disease; NSTEMI, Non-ST elevation myocardial infarction

Multivariable logistic regression analysis was used to identify factors relating to CABG. All statistically different variables (p < 0.1) in Table 1 were entered into the model. The variables that were included by this indication were: hyperlipidemia, prior PCI, prior MI, congestive heart failure, the number of vessels with CAD, on-site cardiac surgery, and the time of enrollment into the registry. Age and gender (pre-specified) were also included in the model due to their clinical significance. Survival analysis was carried out using the Kaplan–Meier method, and comparison by the revascularization strategy (CABG vs. PCI) was tested using the log-rank test. Landmark analysis was performed following the detection of risk reversal in the univariate analysis, as a result of the non-proportional hazard function of the survival curves between PCI and CABG. Furthermore, mortality was evaluated by an additional model for the overall study period based on weighted Cox regression, in order to estimate the average hazard ratio in the event of non-proportional hazard. Statistically significant variables by univariable analysis were used in the multivariable model to identify independent predictors of 10-year mortality. The variables included in the final model were: age, revascularization strategy, gender, hypertension, smoking status, renal impairment, previous stroke and congestive heart failure.

Statistical significance was assumed when the null hypothesis could be rejected at p < 0.05. All p-values reflect results of two-sided tests. Statistical analyses were conducted using R (version 3.4.1).

## Results

### Baseline clinical characteristics

Of the 1987 patients included in the study, 1652 (83%) underwent PCI and 335 (17%) underwent CABG. Mean age of the study patients was 67 ± 11 years, and 30% of the participants were women. Patients treated by PCI had a higher mean left ventricle ejection fraction, higher incidence of prior MI or PCI, with less incidence of three-vessel CAD (Table 1). After propensity score matching, baseline clinical characteristics of study patients, according to their revascularization strategy, presented no statistically significant differences (Table 1).

Indications for the index coronary angiography in all study patients were as follows: NSTEMI in 70%, and unstable angina pectoris in 30%. Among the matched groups the indications were as follows: NSTEMI in 76% and UA in 24%, without statistically significant differences between those who underwent either PCI or CABG (Table 1).

### Factors associated with referral for PCI or CABG

**Fig. 2:**
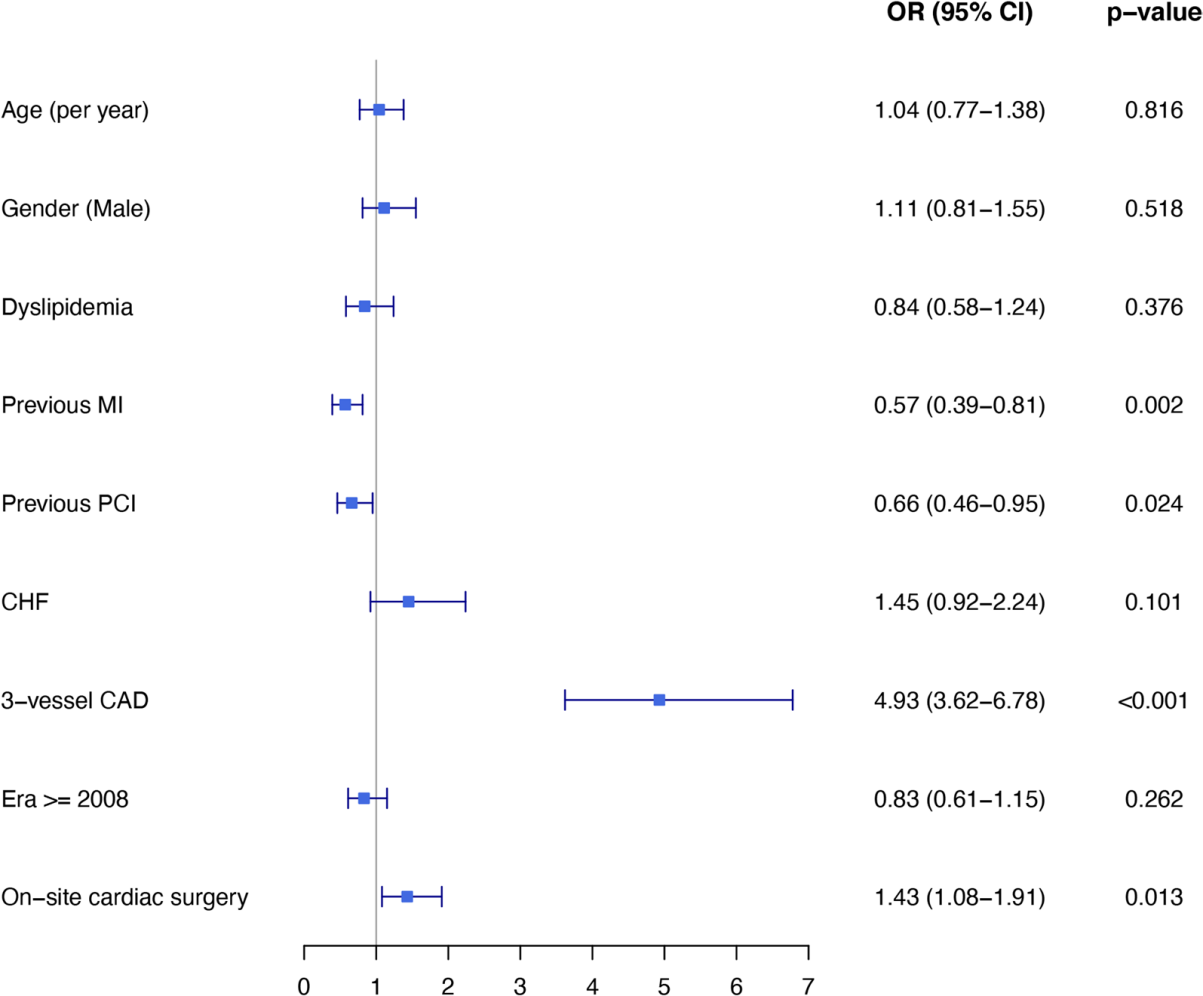
Multivariable logistic regression: Odds ratio for CABG treatment (vs. PCI) with 95% Confidence interval. Patients with multi-vessel disease and those referred from centers with on-site cardiac surgery units, were more likely to be referred to CABG, while those with prior PCI or a previous MI were more likely to have been referred to PCI. Older age, gender and history of congestive heart failure were not associated with any particular revascularization strategy referral pattern. CABG, Coronary artery bypass graft; PCI, Percutaneous coronary intervention; MI, Myocardial infarction; CHF, Congestive heart failure; CAD, Coronary artery disease; OR, Odds ratio; CI, Confidence interval

Multivariable logistic regression analysis showed that 3-vessel CAD vs. 1 or 2-vessel CAD was the most powerful predictor for CABG referral. This analysis showed that patients with 3-vessel CAD were 4.9 times more likely to be referred to CABG, compared with patients who had only 1–2 vessel CAD (p < 0.001). Additional independent predictors for PCI vs. CABG included the absence of an on-site cardiac surgery unit, previous PCI and previous MI. Older age, gender, dyslipidemia, congestive heart failure or revascularization era were not associated with any revascularization strategy referrals (Fig. 2).

### Early outcomes

Early outcomes among the unmatched and matched patients are presented in Table 2. Among the matched patients, those who underwent CABG as opposed to PCI, had a lower rate of recurrent MI at 30 days (0.5% vs. 6%, p = 0.005), and a lower rate of MACE defined as mortality, recurrent MI or stroke at 30 days (3% vs. 10.6%, p = 0.011), with no difference in 30-day (3% vs. 2%, p = 0.755) or 1-year mortality (8% vs 9.6%, log-rank p = 0.580).

**Table 2:** Early results of the unmatched and matched cohort

	Before matching			After matching		
	PCI N = 1652 (%)	CABG N = 335 (%)	p-value	PCI N = 200 (%)	CABG N = 200 (%)	p-value
30-day outcomes						
Mortality	35 (2.1)	11 (3.3)	0.272	6 (3)	4 (2)	0.755
Recurrent MI	47 (3.0)	2 (0.7)	0.031	12 (6)	1 (0.5)	0.005
Stent thrombosis	9 (0.7)	–	–	0 (0)	–	–
CVA	10 (0.6)	0 (0)	0.315	4 (2)	0 (0)	0.132
MACE^a^	89 (7.1)	14 (6.1)	0.704	18 (10.6)	5 (3)	0.011
1-year mortality	125 (7.6)	32 (9.6)	0.264	16 (8)	19 (9.6)	0.712

CABG, Coronary artery bypass graft; PCI, Percutaneous coronary intervention; MI, Myocardial infarction; CVA, Cerebrovascular accident; MACE, Major adverse cardiac events

^a^MACE is defined as mortality, recurrent MI or stroke at 30 days

### Long-term survival

**Fig. 3:**
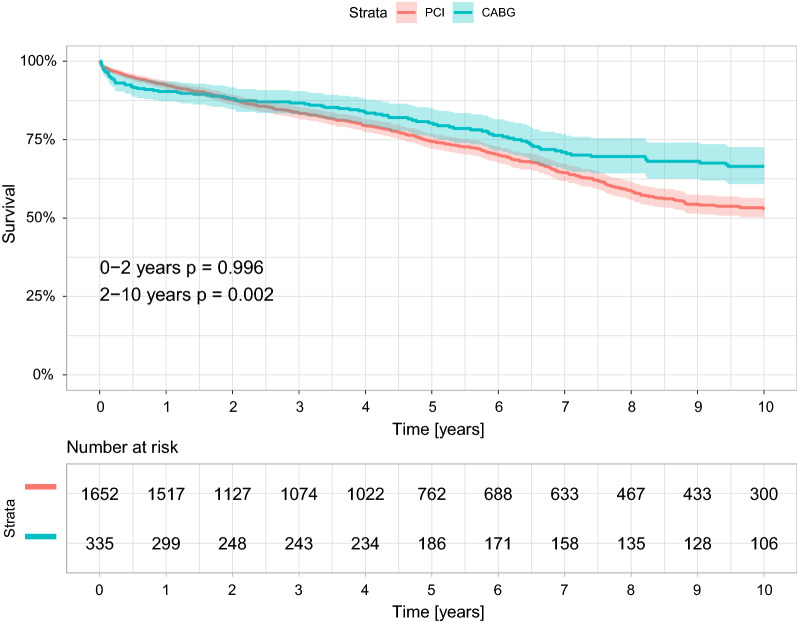
Overall 10-year survival curves by revascularization strategy (unmatched population) *. *p-value is for the landmark analysis: 0–2 years; from 2 years and thereafter. CABG, Coronary artery bypass graft; PCI, Percutaneous coronary intervention

**Fig. 4:**
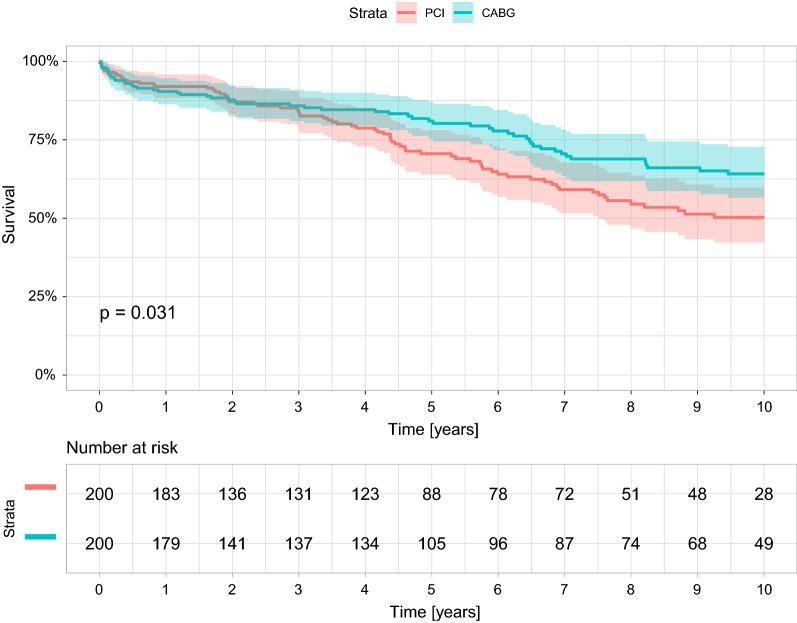
Kaplan Meier curves for survival of the matched pairs. The 10-year mortality hazard was significantly lower in patients who underwent CABG compared to PCI (27% vs. 36%, log-rank p = 0.031). CABG, Coronary artery bypass graft; PCI, Percutaneous coronary intervention

Unadjusted comparison between the two revascularization strategies in the entire unmatched study cohort showed a long-term advantage toward CABG, with a statistically significant treatment-by-time interaction effect (Fig. 3). Thus, 2-year cumulative survival rates were similar in patients who underwent either CABG or PCI (86.9% vs. 87.2%, p = 0.996), whereas landmark analysis showed that beginning with the second year following the intervention, subsequent cumulative survival rates were significantly lower among those who underwent PCI compared to those who had undergone CABG (66.4% vs. 73.1%, p = 0.002) (Fig. 3). Adjusted comparison between the two revascularization strategies by propensity matching analysis showed a long-term advantage toward CABG (Fig. 4). The 10-year mortality hazard was significantly lower in those who underwent CABG compared to PCI (27% vs. 36%, log-rank p = 0.031).

Consistent with these findings, non-proportional multivariable analysis showed that CABG was independently associated with a significant 35% reduction in the risk of 10-year mortality, compared with PCI (p = 0.021). Additional predictors of 10-year mortality included age > 65 years (p = 0.002) and a history of congestive heart failure (p = 0.006) (Table 3).

**Table 3:** Cox regression analysis—predictors for 10-year all-cause mortality

	HR	95% CI	p-value
Age > 65 years	1.81	1.24–2.66	0.002
PCI vs. CABG	1.53	1.07–2.21	0.021
Gender (male)	1.09	0.74–1.63	0.636
Hypertension	1.08	0.68–1.71	0.743
Current smoker	0.95	0.63–1.45	0.823
Renal impairment	1.36	0.89–2.08	0.156
Previous stroke	1.60	0.98–2.60	0.058
History of CHF	1.88	1.20–2.95	0.006

PCI, Percutaneous coronary intervention; CABG, Coronary artery bypass graft; CHF, Congestive heart failure; HR, Hazard ratio; CI, Confidence interval

### Subgroup analyses

A subanalysis showed that the association between CABG and improved outcome was significant in both insulin- and oral antiglycemic treated patients (Additional file 2: Fig. S1 and Additional file 3: Fig. S2), hypertensive and non-hypertensive patients, those with and without congestive heart failure, and regardless to the number of vessels involved (Additional file 4: Fig. S3). Additional analysis demonstrated that CABG provides a greater survival advantage in male sex (Additional file 5: Fig. S4), older patients, patients with no history of renal impairment, and those with previous MI. While all these subgroups were statistically significant, the complementary subgroups showed a trend toward survival advantage for CABG, but this did not reach statistical significance (Additional file 4: Fig. S3 and Additional file 6: Fig. S5).

Among the 1374 patients with NSTEMI, 1140 underwent PCI (83%) and 234 CABG (17%). While there was no significant difference between the two revascularization strategies in the sort-term (Additional file 1: Table S1), long-term survival was significantly higher following CABG (Additional file 7: Fig. S6).

## Discussion

Our study investigated the outcomes of revascularization in diabetic patients with NSTEMI/UA enrolled in a nationwide biennial registry. First, we found that revascularization by CABG revealed excellent long-term outcomes among diabetic patients in a real-life setting. Second, our principal finding was that the advantage of CABG over PCI was seen only at 2 years of follow-up and thereafter. Furthermore, patients with multi-vessel disease and those referred from centers with on-site cardiac surgery units, were more likely to be referred to CABG; while those with prior PCI or a previous MI were more likely to have been referred to PCI. Older age, gender and history of congestive heart failure were not associated with any particular revascularization strategy referral pattern.

While in the general population diabetes is associated with excess mortality compared with non-diabetic patients (with a hazard ratio of 1.15 at 5-years) [12], we report on a greater impact of diabetes on patients who undergo revascularization after NSTEMI/UA (hazard ratio of 1.91 at 5 years). Even though we lacked data regarding the cause of death, MI, and graft patency during the follow-up period, we assumed that this higher impact of diabetes in our cohort, compared to the natural history of diabetes in the general population, was due to accelerated CAD. This assumption was not only based on previous studies that support this theory [13], but also on the recognized fact that diabetes is associated with a high target lesion failure rate [14].

We reported that among diabetic patients presenting with ACS without ST elevation, the 5-year mortality rate was 21.7% in those who underwent revascularization by PCI. Likewise, Farkouh et al. [2] reported a 5-year mortality rate of 26.6% in diabetic patients who underwent PCI, Contini et al. [15] reported a 5-year mortality rate of 24.5%, Kappetein et al. [3] reported a 5-year mortality rate of 19.5%, and Ramanathan et al. [16] reported a 5-year mortality rate of 22.3% in a similar population cohort. Those patients who underwent CABG in our current study had a 5-year mortality rate of 17.9%, which is lower than that published for revascularization by PCI in other series [2, 3, 15, 16]. The survival benefit of CABG in diabetic patients, who often exhibit a diffuse pattern of CAD, characterized by a high burden of atherosclerosis in the entire vasculature, is explained by the fact that CABG frequently achieves more complete revascularization, by providing distal flow to an entire coronary distribution, compared with PCI that generally addresses focal rather than diffuse coronary artery stenoses. Furthermore, the patency of stents is not as effective in diabetic as it is in non-diabetic patients, with a 2–4 times higher risk of developing in-stent restenosis after PCI compared to non-diabetic patients [17, 18]. In addition, it has been shown that diabetic patients demonstrate poorer outcomes following PCI than non-diabetic patients. Even in the era of drug-eluting stents, the rate of repeat revascularizations still remains high in diabetic patients [19].

Diabetes mellitus is one of the chief causes of heart failure, either secondary to CAD or secondary to diabetic cardiomyopathy [20]. It may be related to the impact of a procoagulant imbalance, chronic exposure to high glucose levels, and direct effects of hyperinsulinemia. Interestingly, endogenous hyperinsulinemia has been associated with increased long-term mortality following MI in non-diabetic patients [21]. Further studies are needed to investigate whether insulin-dependent diabetic patients should be included in risk stratification algorithms for patients who undergo revascularization.

During the last decade we have seen a decline in the rate of isolated CABG [22]. In clinical practice physicians tend to under-use surgery despite the fact that candidates are considered suitable.

**Fig. 5:**
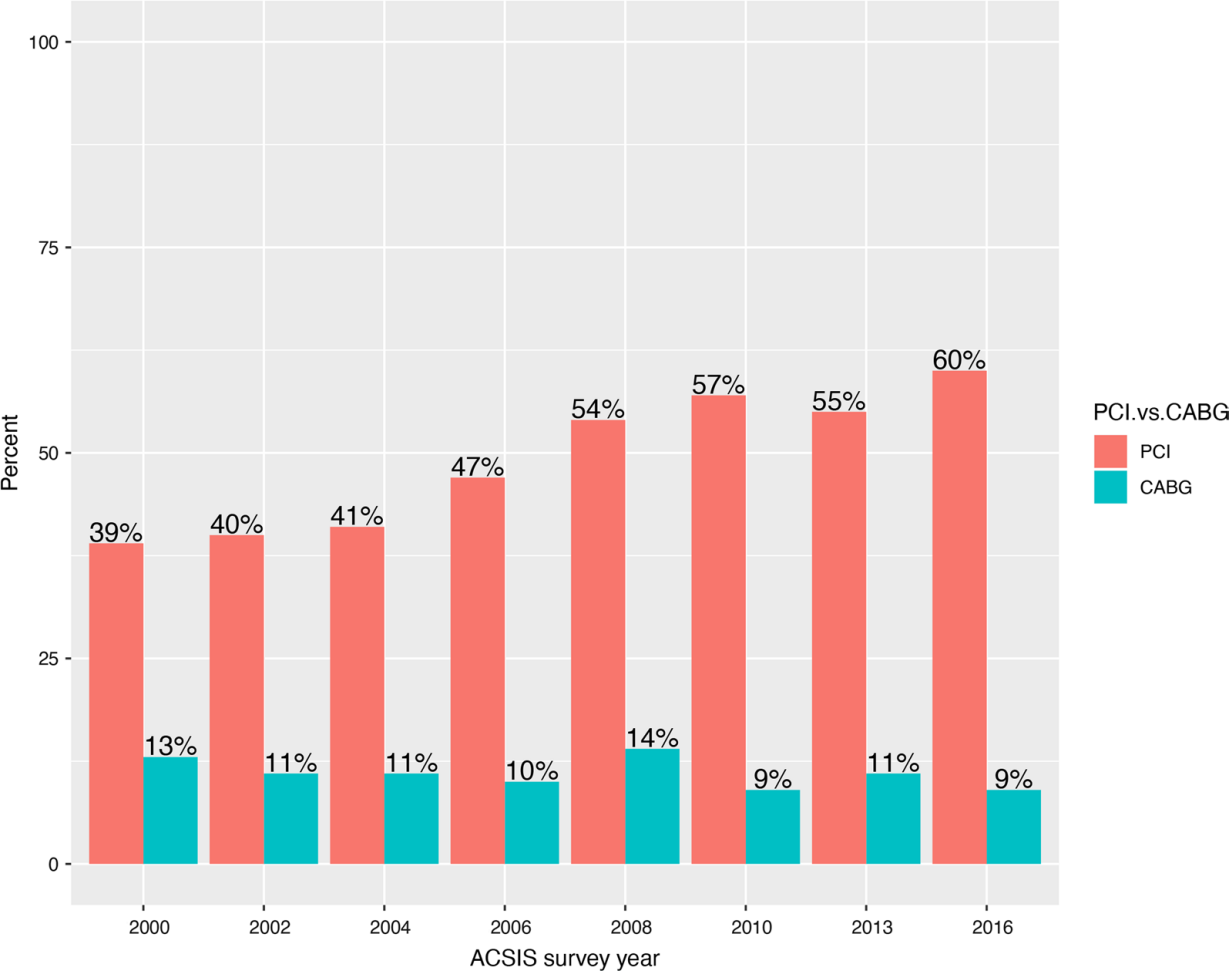
A revascularization pattern throughout the follow-up period for patients with diabetes mellitus and acute coronary syndrome without ST-elevation myocardial infarction. PCI, Percutaneous coronary intervention; CABG, Coronary artery bypass graft; ACSIS, Acute Coronary Syndrome Israeli Survey

Hemingway et al. showed that 26% of patients who demonstrated appropriate indications for CABG, were eventually treated medically, an approach that resulted in adverse clinical outcomes [23]. Furthermore, in the ACSIS registry between 2000 and 2016, there was an increase in the use of PCI for diabetic patients (from 39% in 2000 to 60% in 2016), while the rate for CABG decreased during those years (from 13% in 2000 to 9% in 2016) (Fig. 5). Our current report, however, reinforces the use of CABG in those diabetic patients considered to be eligible candidates.

We have shown that the absence of an on-site cardiac surgery unit was an independent predictor for PCI referral (vs. CABG). Previous studies have reported on inappropriate use of revascularization strategies with lack of case discussion, with a rate of inappropriate use of PCI range between 10 and 15% [24, 25] and inappropriate CABG 1–2% [26]. A heart team discussion on a regular basis may minimize specialty bias and prevent inappropriate revascularization [27]. The findings of this study further emphasize the clinical importance of a multidisciplinary discussion by a heart team approach.

In a previous publication by our group [28], we studied the outcomes of all patients hospitalized with NSTEMI or UA who participated in the ACSIS national registry. We showed that in a real-life setting, revascularization by CABG (vs. PCI) was associated with a survival benefit only in male patients, and was seen only after 4 years and thereafter. The current study is a sub-study also based on data from the ACSIS registry, which compares the two revascularization strategies among diabetic patients. Since the current guidelines for the management of ACS without STEMI among diabetic patients are based predominantly on the results of patients with stable CAD, due to the lack of randomized studies in NSTEMI/UA patients [4], the comparison between the two revascularization strategies is based on cumulative data, thereby justifying the need to analyze the real-life results of diabetic patients who are potential candidates for either one of the revascularization strategies. Furthermore, although the current guidelines are based on the results of patients without ACS, they may not reflect real-life management and the results of NSTEMI/UA diabetic patients.

In accord with previous studies, our study cohort also included predominantly male patients who underwent CABG, with only 26.3% female patients [29–31]. The explanation for this phenomenon could be that cardiovascular disease develops in an older age in females than in males, and older age increases surgical risk. Differences in clinical presentation in women can affect the decision-making process and lead to less aggressive treatment strategies with less referrals for surgical revascularization [32].

### Study limitations

Primarily, the ACSIS registry included patients admitted only to cardiology wards and intensive cardiac care units nationwide, and in the main did not include patients hospitalized in internal medicine wards, thus introducing a selection bias. Data regarding the urgency of the procedure were unavailable. Lack of information regarding the performance of emergency or elective procedures would have been helpful to reduce selection bias between revascularization strategies. There was insufficient anatomical information regarding the complexity of CAD, the specific artery involved, and the surgical techniques performed. Therefore, it is difficult to draw conclusions regarding the association between specific interventions in native arteries or grafts and clinical outcomes. Information was lacking regarding the main cause of death or the rate of cardiac events, such as recurrent revascularization, during the follow-up period. Analysis of cardiac events could have further reinforced the fact that CABG exhibits additional advantages.

## Conclusions

In a real-life setting, revascularization by CABG is associated with good long-term outcomes in diabetic patients with NSTEMI/UA ACS. In light of our preliminary findings, it is hoped that future guidelines will include more effective recommendations based on additional prospective randomized trials regarding the management of diabetic patients hospitalized with NSTEMI or UA, who are deemed eligible for coronary revascularization therapy.

## Supplementary Information

**Additional file 1: Table S1.** Early results of the subgroup of NSTEMI patients.

**Additional file 2: Figure S1.** Overall 10-year survival curves by revascularization strategy among patients treated by insulin. CABG, Coronary artery bypass graft; PCI, Percutaneous coronary intervention.

**Additional file 3: Figure S2.** Overall 10-year survival curves by revascularization strategy among oral antiglycemic treated patients. CABG, Coronary artery bypass graft; PCI, Percutaneous coronary intervention.

**Additional file 4: Figure S3.** Subgroup analysis: HR with 95% CI for 10 years mortality (PCI vs CABG). CABG, Coronary artery bypass graft; PCI, Percutaneous coronary intervention; HR, Hazard ratio; CI, Confidence interval; MI, Myocardial infarction; CAD, Coronary artery disease.

**Additional file 5: Figure S4.** Overall 10-year survival curves by revascularization strategy among subgroup of male patients. CABG, Coronary artery bypass graft; PCI, Percutaneous coronary intervention.

**Additional file 6: Figure S5.** Overall 10-year survival curves by revascularization strategy among subgroup of female patients. CABG, Coronary artery bypass graft; PCI, Percutaneous coronary intervention.

**Additional file 7: Figure S6.** Overall 10-year survival curves by revascularization strategy of patients with non-ST elevation myocardial infarction *. *p-value is for the landmark analysis: 0–2 years; from 2 years and thereafter. CABG, Coronary artery bypass grafting; PCI, Percutaneous coronary intervention.

## Data Availability

Data collected from the ACSIS national registry.

## Abbreviations

CAD: Coronary artery disease
CABG: Coronary artery bypass grafting
PCI: Percutaneous coronary intervention
ACS: Acute coronary syndrome
NSTEMI: Non-ST-segment elevation myocardial infarction
UA: Unstable angina
ACSIS: The Acute Coronary Syndrome Israeli Survey
MACE: Major adverse cardiac events
