# Supplementary material for: Revascularization outcomes in diabetic patients presenting with acute coronary syndrome with non-ST elevation
Source: Cardiovasc Diabetol. 2022 Sep 5;21:175. doi: 10.1186/s12933-022-01595-5 (PMC9443038; doi:10.1186/s12933-022-01595-5)
Supplement: Supplementary file 1 — Additional file 1: Table S1. Early results of the subgroup of NSTEMI patients. [file 12933_2022_1595_MOESM1_ESM.docx]

Additional file

Figure S1:

Overall 10-year survival curves by revascularization strategy among patients treated by insulin.

CABG = Coronary artery bypass graft; PCI = Percutaneous coronary intervention.

Figure S2:

Overall 10-year survival curves by revascularization strategy among oral antiglycemic treated patients.

CABG = Coronary artery bypass graft; PCI = Percutaneous coronary intervention.

Figure S3:

Subgroup analysis: HR with 95% CI for 10 years mortality (PCI vs CABG).

CABG = Coronary artery bypass graft; PCI = Percutaneous coronary intervention; HR = Hazard ratio; CI = Confidence interval; MI = Myocardial infarction; CAD = Coronary artery disease.

Figure S4:

Overall 10-year survival curves by revascularization strategy among subgroup of male patients.

CABG = Coronary artery bypass graft; PCI = Percutaneous coronary intervention.

Figure S5:

Overall 10-year survival curves by revascularization strategy among subgroup of female patients.

CABG = Coronary artery bypass graft; PCI = Percutaneous coronary intervention.

Figure S6:

Overall 10-year survival curves by revascularization strategy of patients with non-ST elevation myocardial infarction *.

* P-value is for the landmark analysis: 0-2 years; from 2 years and thereafter.

CABG = Coronary artery bypass grafting; PCI = Percutaneous coronary intervention.

**Table S1: Early results of the subgroup of NSTEMI patients**

|  | **PCI**  **N = 1140 (%)** | **CABG**  **N= 234 (%)** | **p-value** |
| --- | --- | --- | --- |
| 30-day outcomes |  |  |  |
| Mortality | 33 (2.9) | 9 (3.9) | 0.568 |
| Recurrent MI | 36 (3.2) | 3 (1.3) | 0.177 |
| Stent thrombosis | 7 (0.8) | - | - |
| CVA | 6 (0.5) | 0 (0) | 0.572 |
| MACE * | 81 (8.8) | 12 (6.8) | 0.476 |
| 1-year mortality | 105 (9.3) | 27 (11.6) | 0.320 |

* MACE is defined as mortality, recurrent MI or stroke at 30 days.

NSTEMI = non-ST elevation myocardial infarction; CABG = Coronary artery bypass graft; PCI = Percutaneous coronary intervention; CVA = Cerebrovascular accident; MACE = Major adverse cardiac events.
