# Supplementary figures and images for: Revascularization outcomes in diabetic patients presenting with acute coronary syndrome with non-ST elevation
Source: Cardiovasc Diabetol. 2022 Sep 5;21:175. doi: 10.1186/s12933-022-01595-5 (PMC9443038; doi:10.1186/s12933-022-01595-5)

Strata — PCI — CABG

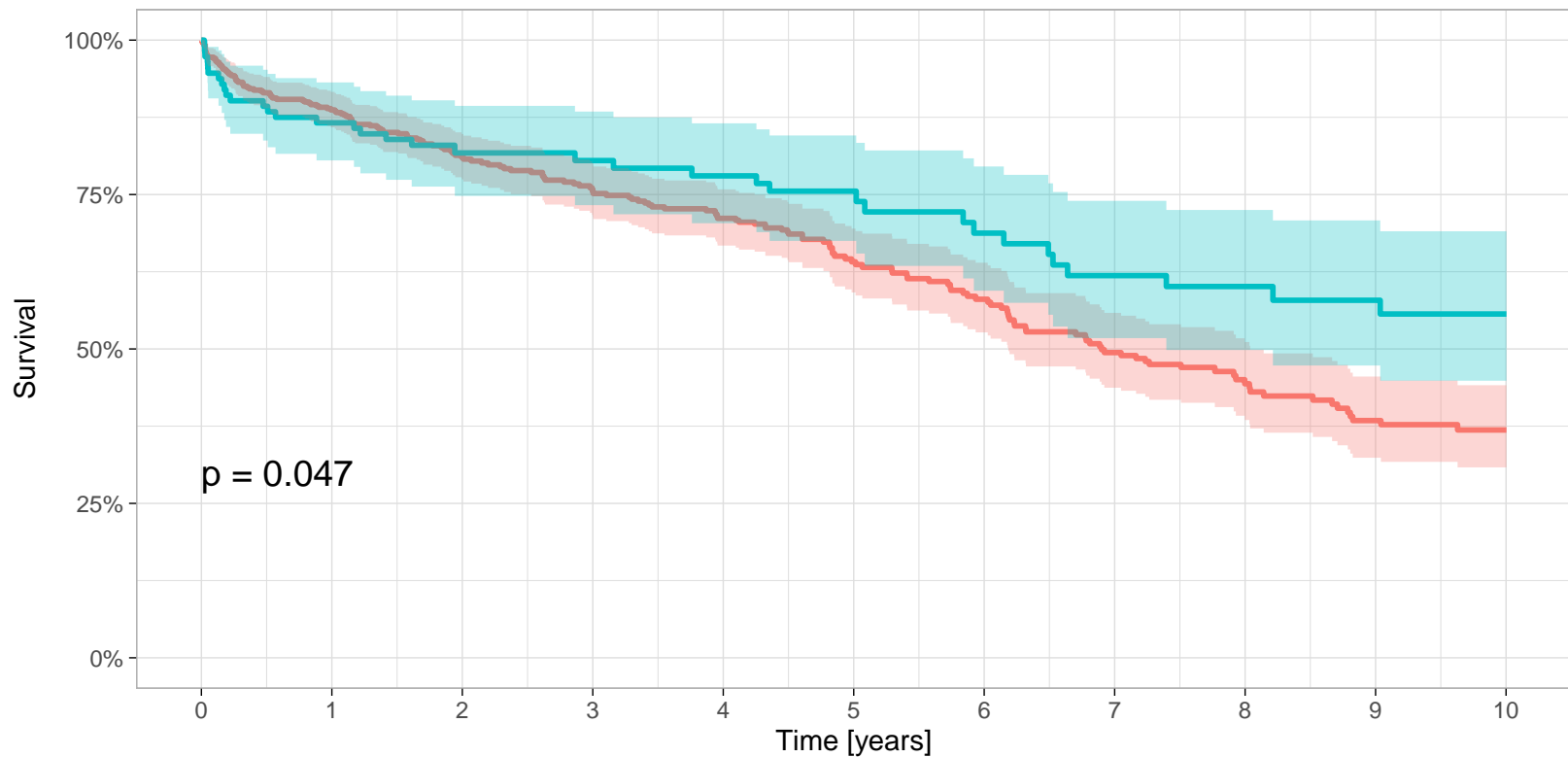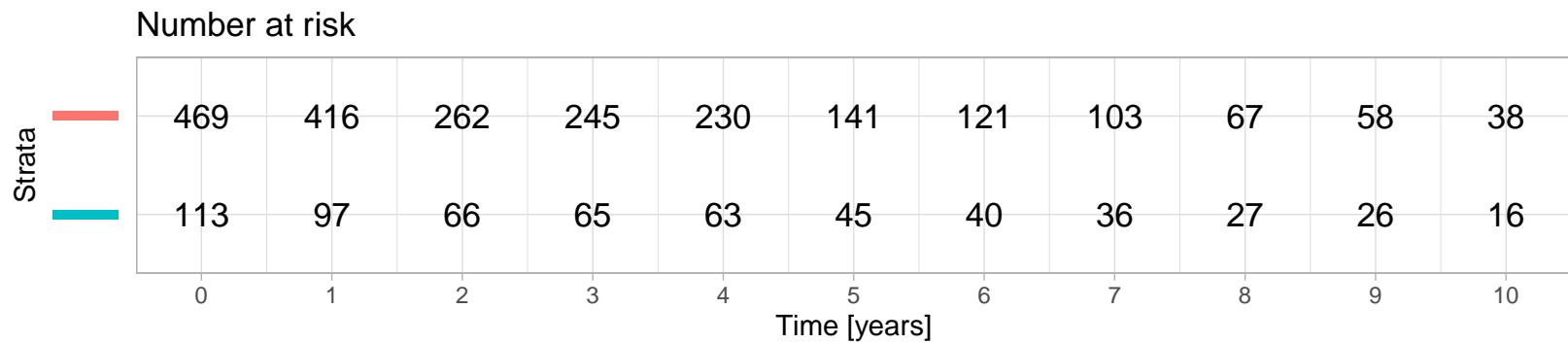

Supplement: Supplementary file 2 — Additional file 2: Figure S1. Overall 10-year survival curves by revascularization strategy among patients treated by insulin. CABG, Coronary artery bypass graft; PCI, Percutaneous coronary intervention. [file 12933_2022_1595_MOESM2_ESM.pdf]

Strata    PCI    CABG

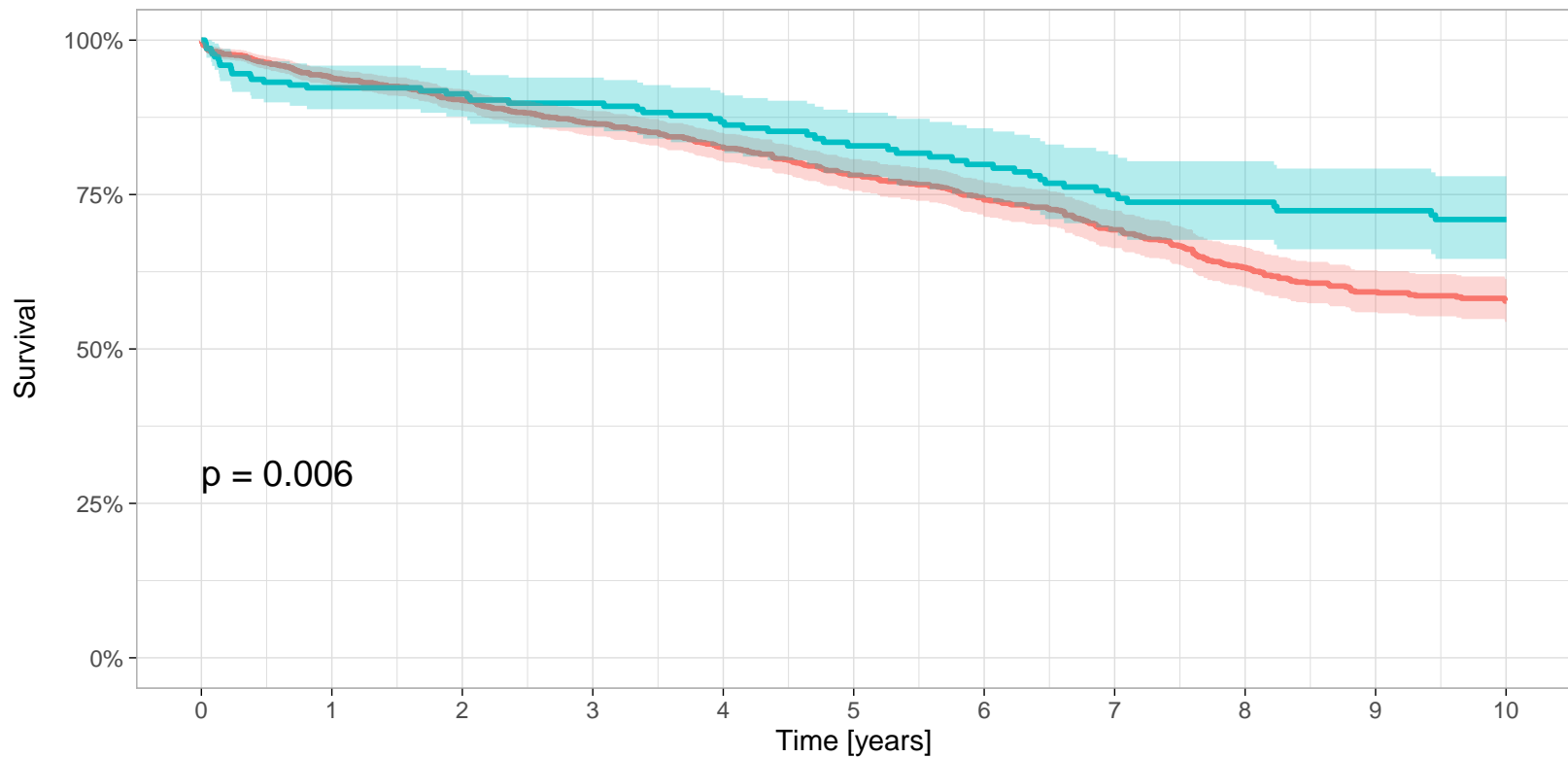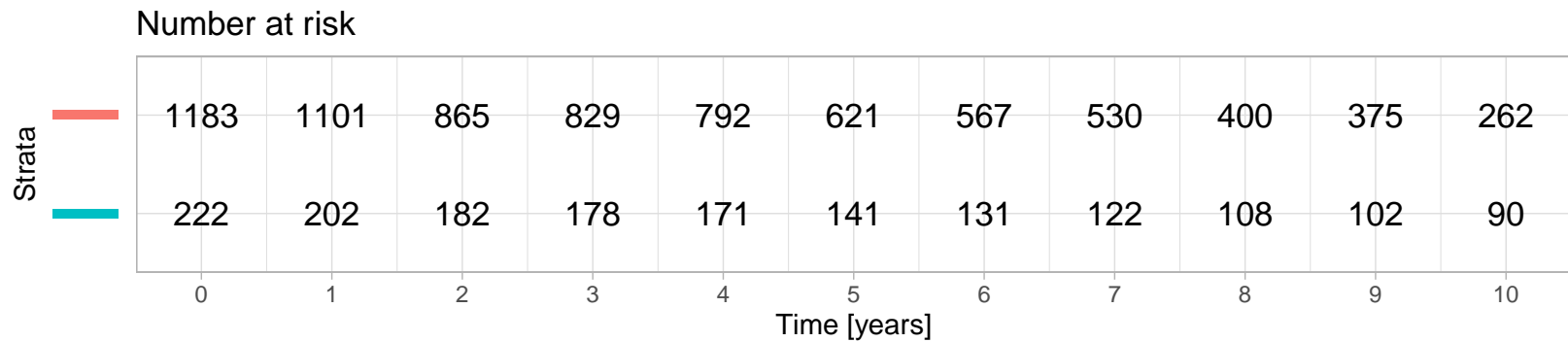

Supplement: Supplementary file 3 — Additional file 3: Figure S2. Overall 10-year survival curves by revascularization strategy among oral antiglycemic treated patients. CABG, Coronary artery bypass graft; PCI, Percutaneous coronary intervention. [file 12933_2022_1595_MOESM3_ESM.pdf]

## HR with 95% CI for late mortality (PCI vs. CABG)

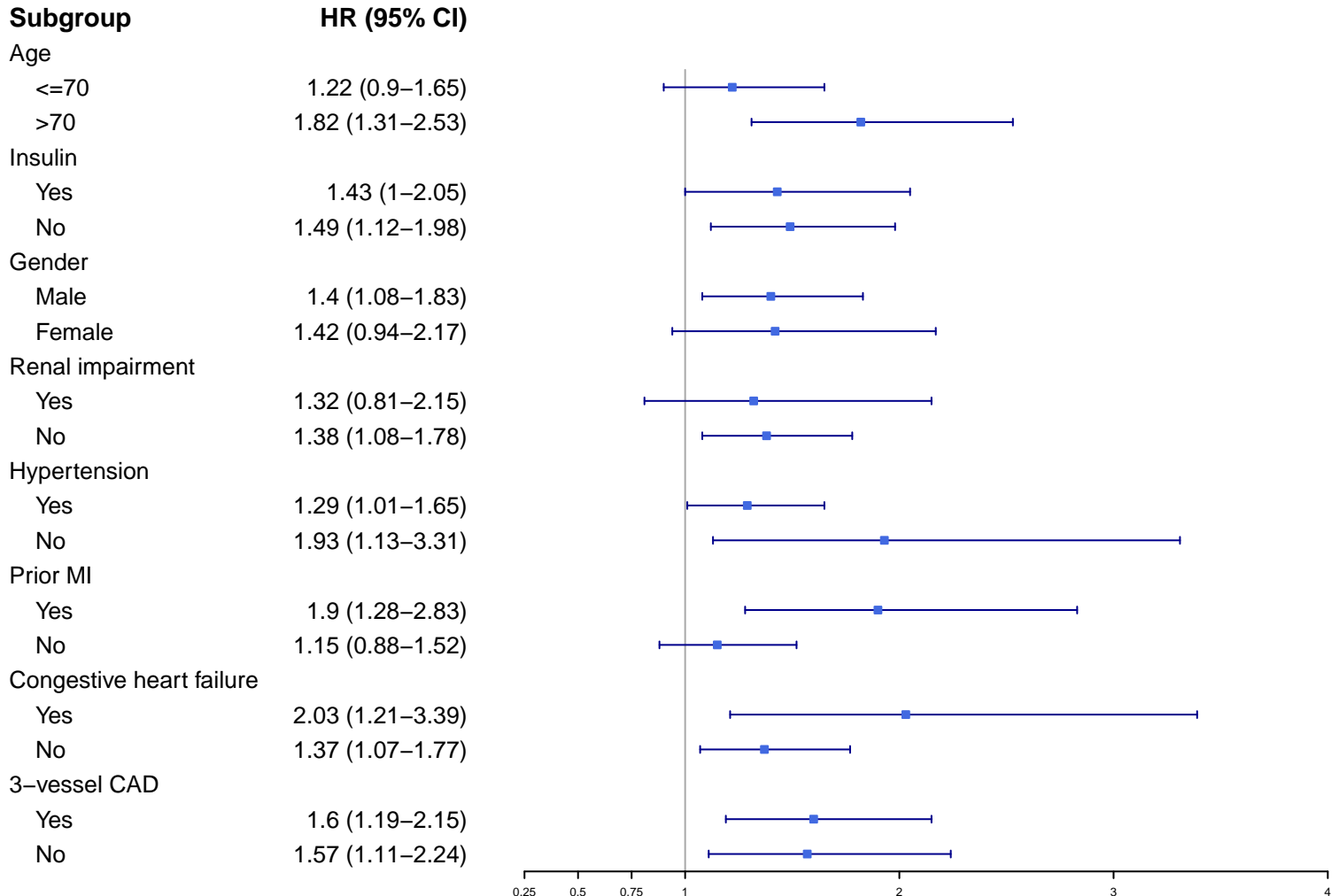

Supplement: Supplementary file 4 — Additional file 4: Figure S3. Subgroup analysis: HR with 95% CI for 10 years mortality (PCI vs CABG). CABG, Coronary artery bypass graft; PCI, Percutaneous coronary intervention; HR, Hazard ratio; CI, Confidence interval; MI, Myocardial infarction; CAD, Coronary artery disease. [file 12933_2022_1595_MOESM4_ESM.pdf]

Strata    PCI    CABG

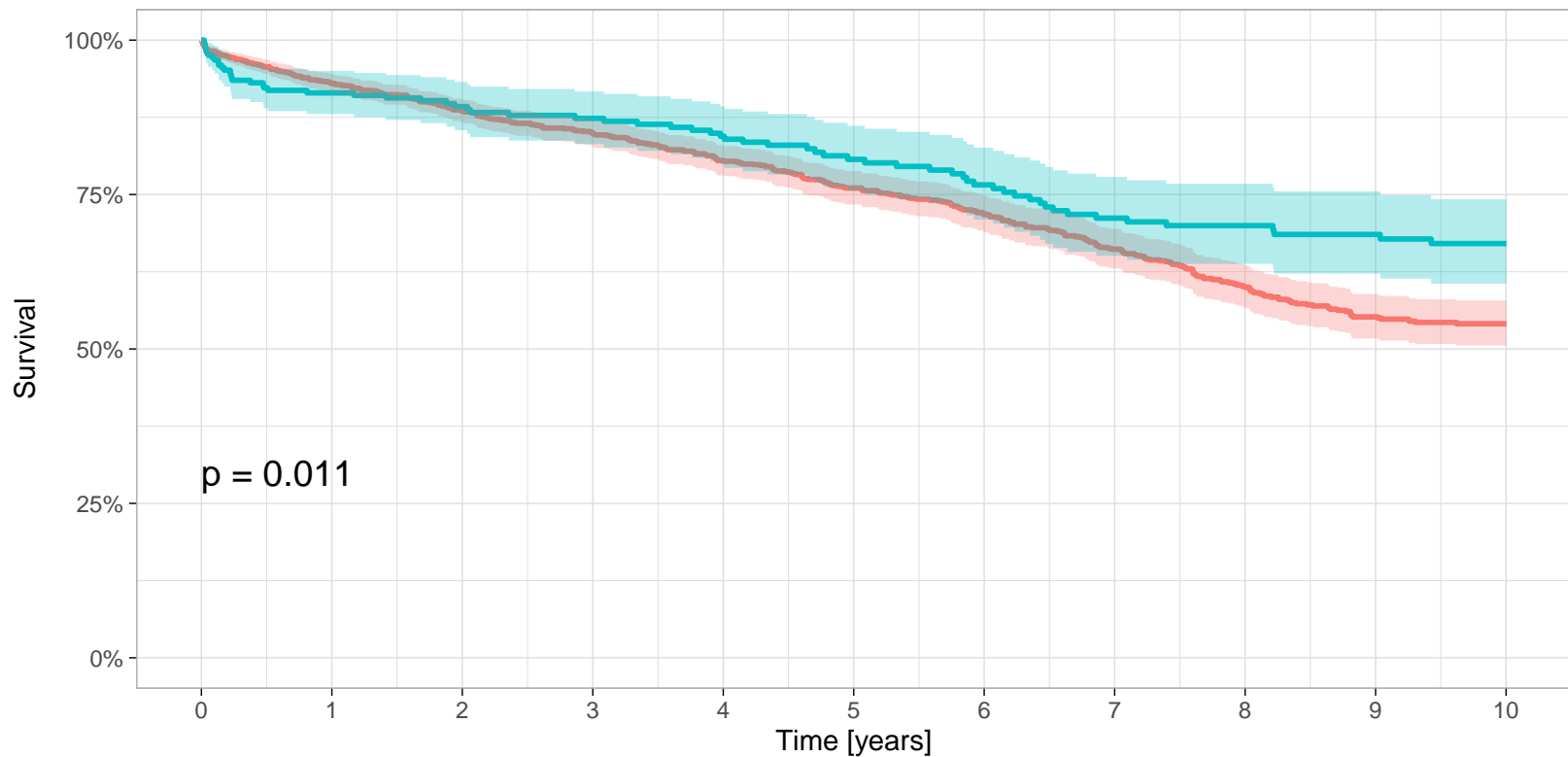

Number at risk

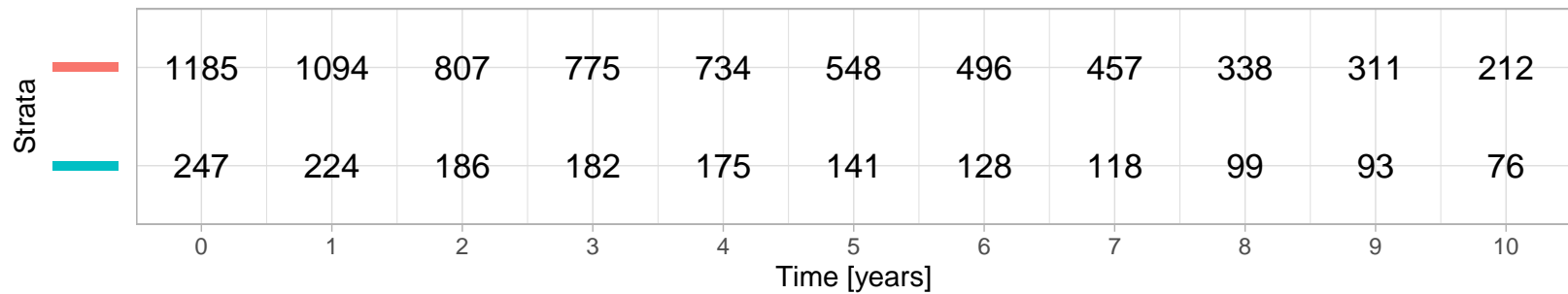

Supplement: Supplementary file 5 — Additional file 5: Figure S4. Overall 10-year survival curves by revascularization strategy among subgroup of male patients. CABG, Coronary artery bypass graft; PCI, Percutaneous coronary intervention. [file 12933_2022_1595_MOESM5_ESM.pdf]

Strata    PCI    CABG

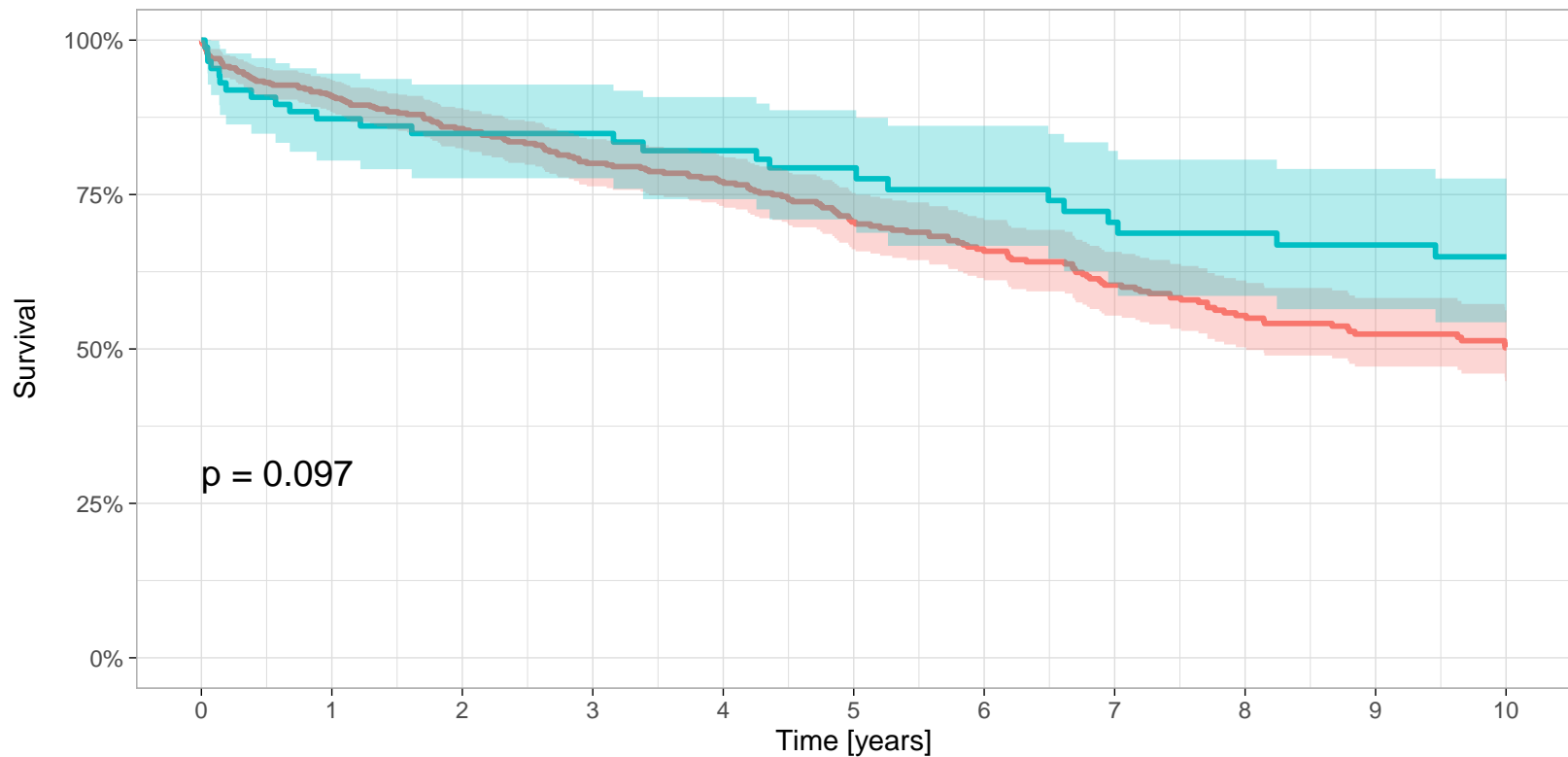

Number at risk

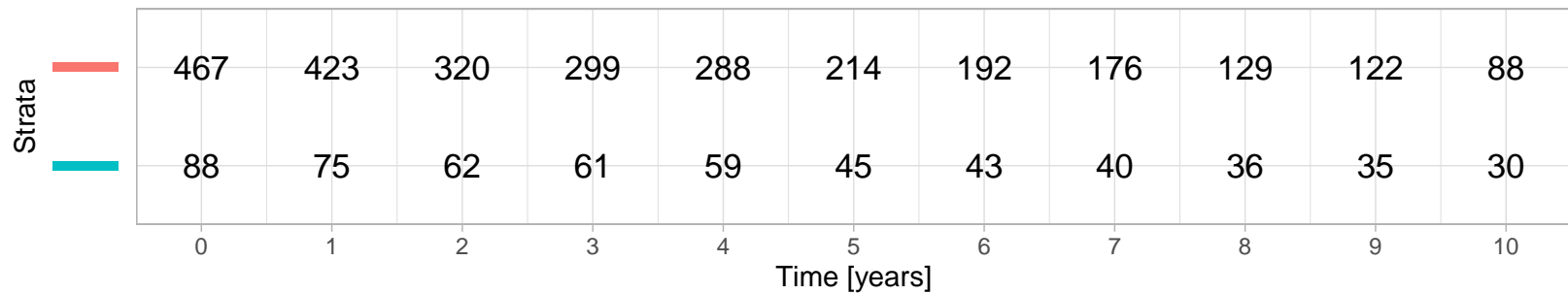

Supplement: Supplementary file 6 — Additional file 6: Figure S5. Overall 10-year survival curves by revascularization strategy among subgroup of female patients. CABG, Coronary artery bypass graft; PCI, Percutaneous coronary intervention. [file 12933_2022_1595_MOESM6_ESM.pdf]

Strata    PCI    CABG

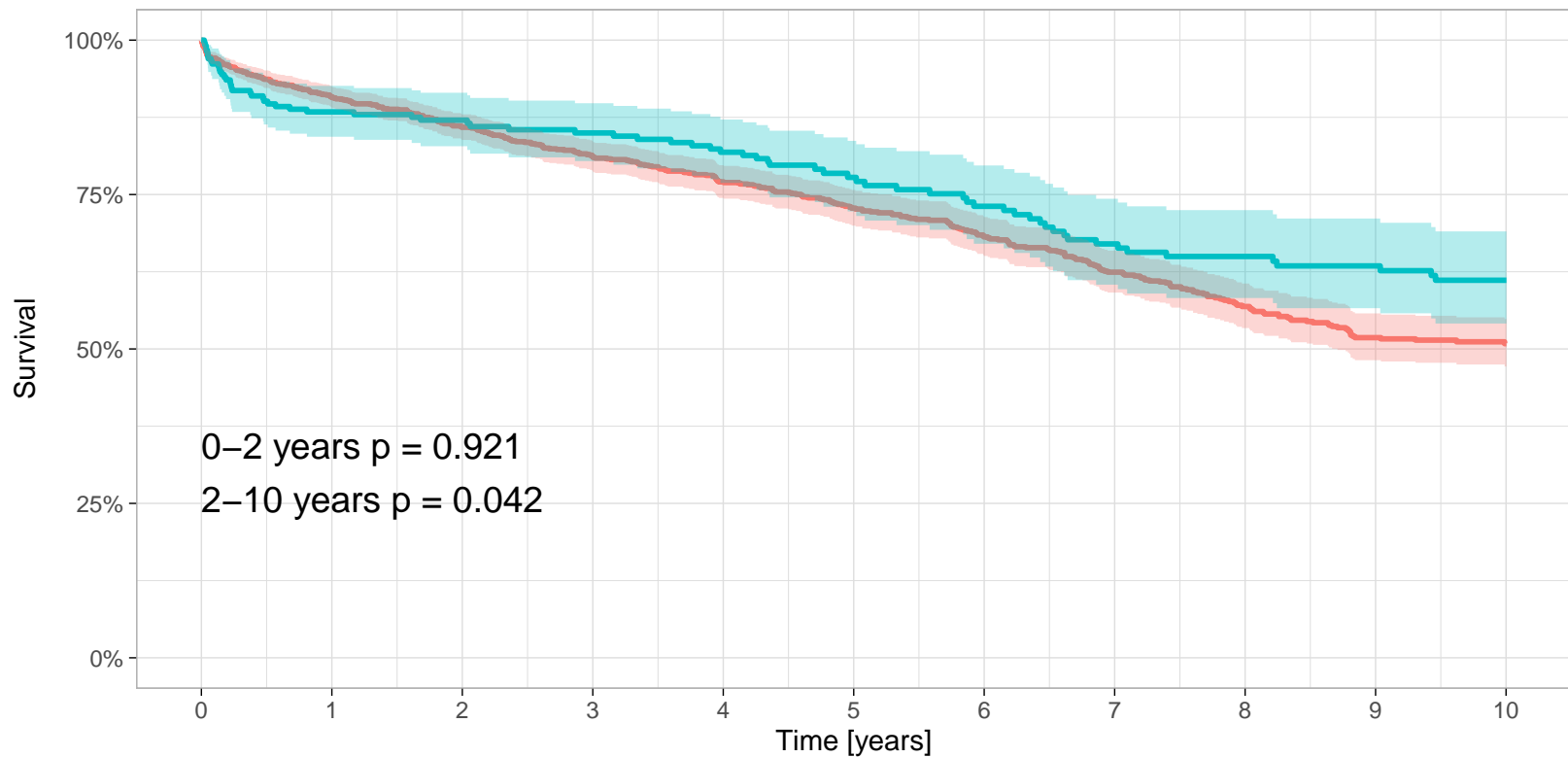

Number at risk

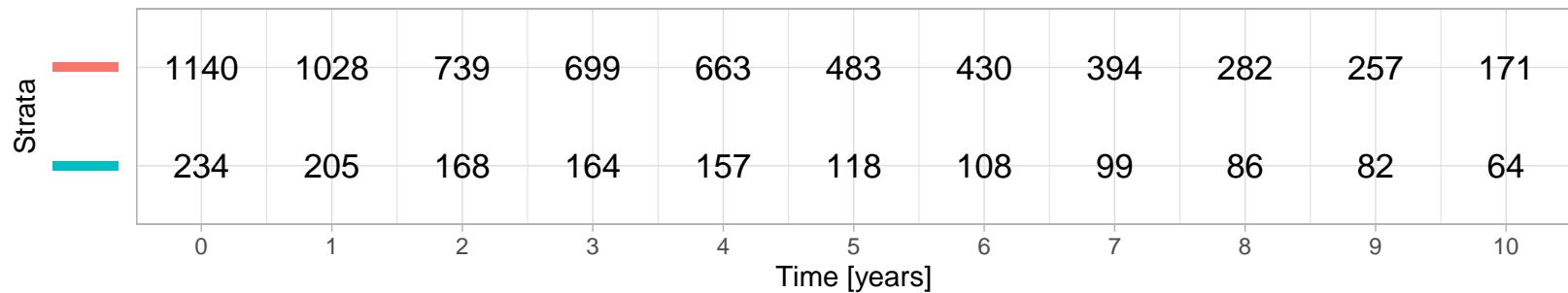

Supplement: Supplementary file 7 — Additional file 7: Figure S6. Overall 10-year survival curves by revascularization strategy of patients with non-ST elevation myocardial infarction *. *p-value is for the landmark analysis: 0–2 years; from 2 years and thereafter. CABG, Coronary artery bypass grafting; PCI, Percutaneous coronary intervention. [file 12933_2022_1595_MOESM7_ESM.pdf]
